# Vector Competence for DENV-2 Among *Aedes albopictus* (Diptera: *Culicidae*) Populations in China

**DOI:** 10.3389/fcimb.2021.649975

**Published:** 2021-03-23

**Authors:** Yong Wei, Jiatian Wang, Yuan-Huan Wei, Zhangyao Song, Ke Hu, Yulan Chen, Guofa Zhou, Daibin Zhong, Xueli Zheng

**Affiliations:** ^1^ Department of Pathogen Biology, School of Public Health, Southern Medical University, Guangzhou, China; ^2^ Department of Clinical Nutrition, Huazhong University of Science and Technology Union Shenzhen Hospital, Shenzhen, China; ^3^ Program in Public Health, College of Health Sciences, University of California, Irvine, Irvine, CA, United States

**Keywords:** *Aedes albopictus*, dengue, vector competence, immune genes, *Wolbachia*

## Abstract

*Aedes albopictus* is a vector of over 20 arboviruses that has spread throughout the world, mainly in the second half of the twentieth century. Approximately 50–100 million people are infected with dengue virus (DENV) transmitted by *Aedes* mosquitoes each year, leading to heavy economic burdens for both governments and individuals, among countless other negative consequences. Understanding the vector competence of vector species is critical for effectively preventing and controlling vector-borne diseases. Accordingly, in this study, vector competence was evaluated by quantitative analysis of DENV-2 loads in mosquito tissues (midguts, heads, and salivary glands) and whole mosquitoes through real-time quantitative polymerase chain reaction (RT-qPCR) analysis. *Wolbachia* and the expression of immune-associated genes (Rel1, Rel2, Dicer2, and STAT) in mosquitoes were also detected by RT-qPCR to explore their impact on vector competence. The amount of DENV-2 in the mosquito midguts, heads, and salivary glands from southern-western China were found to be lower than those from eastern-central-northern China. The DENV-2 loads in whole mosquitoes showed a negative correlation with Rel1 gene (*r* = -0.285, *P* = 0.011) and STAT gene expression levels (*r* = -0.289, *P* = 0.009). In terms of *Wolbachia* strains, the density of the *w*AlbB strain was found to be significantly higher than that of the *w*AlbA strain in the eight *Ae. albopictus* populations, and the relative density of the *w*AlbB strain in mosquitoes from southern-western China was higher than those from eastern-central-northern China. The relative density of the *w*AlbB strain showed a negative correlation with the mean loads of DENV-2 in the heads (*r* = -0.729, *P* = 0.040), salivary glands (*r* = -0.785, *P* = 0.021), and whole mosquitoes (*r* = -0.909, *P* = 0.002). Thus, there are lower DENV-2 loads in the mosquitoes from southern-western China, which may be related to the innate immunity of mosquitoes as affected by Rel1 in the Toll pathway, STAT in the JAK-STAT pathway, and the relative density of the *w*AlbB strain.

## Introduction

*Aedes* (*Stegomyia*) *albopictus* (Skuse, 1894) is an anthropophilic mosquito that originated in Southeast Asia and has spread to many countries across all continents except Antarctica (Goubert et al., 2016). This species is a vector for more than 20 arboviruses, of which some are strongly pathogenic and transmissible, such as dengue virus (DENV), Chikungunya virus (CHIKV), and yellow fever virus (Gratz, 2004; Amraoui et al., 2019).

Dengue is an acute infectious disease caused by DENV, which is transmitted by *Aedes* mosquitoes (World Health Organization [WHO], 2009). There are an estimated 390 million dengue infections each year, of which 96 million manifest clinically (Bhatt et al., 2013), among an estimated 2.5 billion to 4 billion people living in over 100 countries where DENV transmission occurs (World Health Organization [WHO], 2009; Brady et al., 2012; Bhatt et al., 2013; World Health Organization [WHO], 2016).

In China, dengue fever and dengue hemorrhagic fever epidemics occur frequently in the southern regions. Prior to 2000, these regions mainly comprised Guangdong, Hainan, Guangxi, and Fujian Provinces (Chen and Liu, 2015). However, dengue fever epidemics have occurred in more northerly areas, including Zhejiang province (in 2005 and 2011) in southeastern China and Henan province (in 2013) in temperate central China, which is far more northerly than the regions of previous epidemics (Lai et al., 2015). Since 2000, dengue epidemics have also been reported frequently in Yunnan province in southwestern China, which borders Myanmar, Laos, and Vietnam (Lai et al., 2015). The most devastating recent dengue fever epidemic occurred in 2014 in Guangzhou, Guangdong province, causing a total of 47,056 clinical cases and six deaths (Lai et al., 2015; Sun et al., 2017). In most of these past epidemics, *Ae. albopictus* was the sole DENV vector.

The vector competence for DENV of *Aedes* mosquitoes is influenced not only by ecological structure, climate, virus titer, and the serotype and virulence of DENV, but also by endosymbiont bacteria and genetic factors of mosquitoes (Rosen et al., 1983; Cox et al., 2011; Xiao et al., 2014; Méndez et al., 2015; Tsai et al., 2017). Accordingly, the susceptibilities to dengue virus of different mosquito species are significantly different, as are those of different geographic strains of the same mosquito species.

Laboratory colonies of *Ae. albopictus* strains from China have been used to assess vector competence for several dengue virus strains, such as the DEN2-43 and New Guinea C virus strains of dengue-2 viruses (Guo et al., 2013), as well as the DEN2-FJ10 and DEN2-FJ11 strains of dengue virus (Guo et al., 2016). However, relatively little is known about the vector competences of natural populations.

Studies on vector susceptibility to pathogens and vector competence have revealed their strong genetic basis (Gooding, 1996). However, laboratory-colonized mosquito populations suffer from inbreeding depression under laboratory conditions and are subject to a completely different set of selective pressures compared to natural populations (Leftwich et al., 2015). For example, Vazeille et al. (2003) reported a significant increase in the infection rate of *Ae. albopictus* by a DEN2 virus strain with increasing generations in the laboratory; while significant variations in susceptibility to four dengue serotypes was observed among 13 geographic strains of *Ae. albopictus* (Gubler and Rosen, 1976) and 13 geographic strains of *Ae. aegypti* (Gubler et al., 1979). Furthermore, Paupy et al. (2001) found that the populations of *Ae. albopictus* from the east coast of Reunion Island are more susceptible to DENV-2 than those from the west coast.

The innate immune response of the mosquito is a key determinant for successful transmission of mosquito-borne viruses. Viral infection triggers the activation of innate immunity pathways, including the RNA interference (RNAi) pathway, the Janus kinase-signal transducer and activator of transcription (JAK-STAT) pathway, Toll pathway, and immune deficiency (Imd) pathway (Lemaitre and Hoffmann, 2007; Fragkoudis et al., 2008; Xi et al., 2008; Paradkar et al., 2012; Goic et al., 2016; Angleró-Rodríguez et al., 2017), and leads to the transcription of genes responsible for antiviral responses. Rel1, Rel2, Dicer2, and STAT are the key factors in the Toll, Imd, RNAi, and JAK-STAT pathways, respectively (Bian et al., 2005; Antonova et al., 2009; Lambrechts et al., 2013; Kumar et al., 2018; Cabral et al., 2020), and these factors have shown effectiveness against viral infections in some mosquito species (Fragkoudis et al., 2008; Xi et al., 2008; Paradkar et al., 2012; Lambrechts et al., 2013; Angleró-Rodríguez et al., 2017).

The endosymbiotic bacteria *Wolbachia* exists as type A and type B strains in *Ae. albopictus* populations in China (Wei et al., 2019). In mosquitoes, *Wolbachia* can induce cytoplasmic incompatibility (CI) as a means to reduce mosquito populations (Jiggins, 2017) and induce density-dependent inhibition of the dengue virus in mosquito cells (Lu et al., 2012). Accordingly, it has been evaluated for the prevention and control of mosquito-borne diseases in field studies (Hoffmann et al., 2011; Zheng et al., 2019).

In our previous study, we divided the 34 populations of *Ae. albopictus* from northern and southern China into five different genetic clustering groups according to genetic distance (Wei et al., 2019). We then selected eight populations of *Ae. albopictus* from different genetic clusters and performed virus-infection experiments as a means to explore the impact of *Wolbachia* and immune-associated gene expression on dengue susceptibility in the same environment.

The present study is a continuation of that work that aims to address the following questions: (i) What are the vector competences for DENV-2 of different geographic strains of *Ae. albopictus* populations in China? (ii) Is susceptibility to DENV-2 affected by *Wolbachia* and immune-associated genes in *Ae. albopictus* populations? The data gained from this study provides useful information for understanding the epidemiology, prevention, and control of vector-borne diseases.

## Materials and Methods

### Mosquito Sampling

From August to September, 2019, we collected mosquito eggs from different genetic clusters in the eight locations used in our previous study (Wei et al., 2019) (**Table 1**). The eight locations were Beijing (BJ), Shijiazhuang (SJZ), Hangzhou (HZ), Wuhan (WH), Meishan (MS), Guangzhou (GZ), Zhanjiang (ZJ), and Lingshui (LS). In each location, 8–12 oviposition sites 400–3000 m apart were selected randomly for egg sampling. Ovitraps placed under bushes were used for egg collection.

**Table 1:** Information on *Ae. albopictus* collection used in this study.

Sample sites	Abbreviation	Latitude	Longitude	Collection date
Beijing	BJ	39°51’36”N	116°11’45”E	August 2019
Shijiazhuang	SJZ	37°54’55”N	114°27’49”E	August 2019
Hangzhou	HZ	30°18’42”N	120°07’09”E	August 2019
Wuhan	WH	30°30’30”N	114°22’39”E	August 2019
Meishan	MS	30°11’55”N	103°52’01”E	August 2019
Guangzhou	GZ	23°11’15”N	113°19’42”E	September 2019
Zhanjiang	ZJ	21°05’37”N	109°42’60”E	September 2019
Lingshui	LS	18°30’27”N	110°01’59”E	September 2019

Eggs were returned to the laboratory and reared to adults. The larvae were fed daily with turtle food (Shenzhen INCH-GOLD Fish Food Ltd., Shenzhen, China). *Ae. albopictus* adults were identified (Huang and Ward, 1981) and selected for further breeding at 28 ± 1°C with a 16 h:8 h light/dark diurnal cycle at 80 ± 5% relative humidity in 20 cm × 20 cm × 35 cm cages covered with nylon mesh. Mosquito adults were provided with 10% glucose solution.

### Oral Infection of Mosquitoes

DENV-2 virus (New Guinea C strain, GenBank: AF038403.1) was provided by the Key Laboratory of Tropical Disease Control of Sun Yat-sen University (Guangzhou, China). The virus stock was obtained after 5–7 days incubation in C6/36 cells at 28°C and stored at -80°C. The frozen virus stock was passaged once through C6/36 cells before the mosquitoes were infected. A fresh virus suspension was used to prepare the blood meal for oral infection.

The 5–7-day-old and non-blood-fed female mosquitoes were starved for 24 h before infection. The females were then fed with an infectious blood meal (the fresh DENV-2 virus suspension was diluted in defibrinated sheep blood to 5.77 × 10^5^ log_10_ copies/μL). After 30 min of exposure to the infectious blood meal, fully engorged mosquitoes were transferred to a 1500-mL plastic bucket covered with nylon mesh. They were provided with 10% glucose and maintained for 14 days in an HP400GS incubator (Ruihua, Wuhai, China) at 28°C, 80% relative humidity, and a light/dark cycle of 16 h:8 h.

### RNA Extraction and Real-Time Quantitative Polymerase Chain Reaction (RT-qPCR) Analysis

At 14 days post-infection (dpi), 40 female mosquitoes were randomly collected and cold-anesthetized. The legs and wings of 30 mosquitoes were removed, then the heads, salivary glands, and midguts were dissected, washed three times in cold phosphate-buffered saline, and transferred to 50 μL TRIzol (Ambion, Life Technologies, Carlsbad, CA, USA). The remaining 10 whole mosquitoes were placed directly in 50 μL TRIzol after washing in cold phosphate-buffered saline. The total RNA of each tissue or individual was extracted with TRIzol reagent following the manufacturer’s protocol and reverse-transcribed into cDNA using 5× All-In-One MasterMix as supplied with the AccuRT Genomic DNA Removal Kit (Abm, Richmond, Canada). A standard plasmid was constructed for DENV-2 quantification. A 127-bp fragment of the untranslated region (UTR) of DENV-2 was amplified using specific primers (forward: 5’-TCCCTTACAAATCGCAGCAAC-3’; and reverse: 5’-TGGTCTTTCCCAGCGTCAAT-3’) (Liu et al., 2017) and cloned into a pMD18-T vector. After sequencing, the recombinant plasmid was linearized using *EcoR* I. The concentration of the plasmid was detected using a NanoDrop 2000 Spectrophotometer (Thermo Scientific, Wilmington, DE, USA). The 127-bp fragment of the DENV-2 UTR was used as the detecting target for RT-qPCR, and a standard curve was generated by analyzing serial 10-fold dilutions of the plasmid. The viral RNA copies in all of these tissues were quantified by detecting the cDNA of DENV-2 using Hieff^®^ qPCR SYBR^®^ Green Master Mix (YEASEN, Shanghai, China). The RT-qPCR reaction mixture (per well) contained 10 μL SYBR^®^ green master mix, 1 μL of each primer (10 μM), 2 μL cDNA or the plasmid standard, and 6 μL RNase-free water. The reaction was performed in a QuantStudio™ Real-Time PCR System (Applied Biosystems, Foster City, CA, USA) under the following conditions: 95°C for 10 min; 40 cycles of 95°C for 10 s, 60°C for 60 s. DENV-2 RNA copies from each sample were quantified by comparing cycle threshold values with the standard curve.

### Quantification of *Wolbachia* and Immune-Associated Gene Expression Levels in Mosquitoes

Standard plasmids containing target fragments of *Wolbachia* strain A (*w*AlbA) and strain B (*w*AlbB) were used to quantify *Wolbachia* densities in field-collected *Ae. albopictus* using the strain-specific primers qAF/qAR and qBF/qBR (**Table S1**). The expression levels of immune-associated genes in mosquitoes infected with DENV-2 were quantified by comparing cycle threshold values with standard curves from recombinant plasmids containing gene fragments of Rel1, Rel2, Dicer2, and STAT. The quantification of *Wolbachia* and immune-associated gene expression levels in mosquitoes was normalized with the ribosomal protein S6 (Rps6) gene, which was also quantified from recombinant plasmids containing Rps6 fragments. Quantification PCR reactions were performed using solutions with a final volume of 20 µL containing 10 µL of SYBR^®^ green master mix, 0.5 µM of each primer, 2 µL of template DNA, and 7 µL of RNase-free water. The primers of for all the detecting fragments are shown in **Table S1**. The thermal cycling conditions were: 10 min at 95°C; 40 cycles of 95°C for 15 s, primer T_m_ for 30 s (T_m_ values for each primer pair are shown in **Table S1**), 72°C for 30 s.

### Data Analysis

The vector competences of the *Ae. albopictus* mosquitoes were evaluated by calculating the infection rate (IR; no. infected mosquitoes/no. tested mosquitoes), the midgut infection rate (MIR; no. infected midguts/no. tested midguts), dissemination rate (DR; no. infected heads/no. infected midguts), potential transmission rate (TR; no. infected salivary glands/no. infected midguts), and potential population transmission rate (PTR; no. infected salivary glands/no. tested midguts) (Deng et al., 2019). Pearson’s Chi-square tests or Fisher’s exact tests were applied to compare IR, MIR, DR, TR, and PTR values among different geographical strains. Fisher’s exact test was used when the minimum expected count was < 5. The DENV-2 RNA copy levels were log-transformed and then compared among different populations and among the expression levels of immune-associated genes using one-way analysis of variance (ANOVA) *post hoc* Tukey’s LSD tests. Student’s t-test was used to compare the mean DENV loads in mosquitoes between from southern-western China (MS, GZ, ZJ, LS) and from eastern-central-northern China (BJ, SJZ, HZ, WH), and the densities between *w*AlbA and *w*AlbB strains in individual mosquitoes. Pearson’s correlation coefficient was used to analyze the correlation between the average dengue virus load and gene expression in mosquito populations. *P* < 0.05 was considered statistically significant.

## Results

### Vector Competence of *Ae. albopictus* for DENV-2

**Figure 1:**
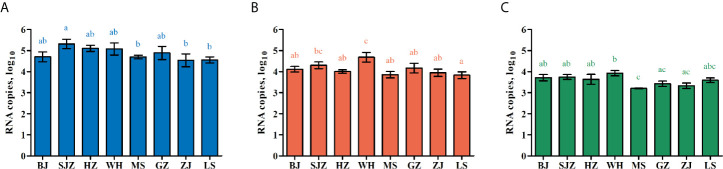
Dengue virus RNA copies in infected midguts **(A)**, heads **(B)**, and salivary glands **(C)** of *Ae. albopictus* in China. The results are expressed as mean ± standard error (SE). Different letters with the same color above bars represent significant differences in relative expression levels at the *P* < 0.05 level.

At 14 dpi, IR, MIR, DR, TR and PTR value for the eight *Ae. albopictus* populations were calculated. The data are shown in **Table 2**. They are not significantly different among the eight population of *Ae. albopictus* (IR: χ^2^ = 4.848, *df* = 7, *P* = 0.710; MIR: χ^2^ = 9.825, *df* = 7, *P* = 0.185; DR: χ^2^ = 5.655, *df* = 7, *P* = 0.583; TR: χ^2^ = 5.936, *df* = 7, *P* = 0.555; PTR: χ^2^ = 4.824, *df* = 7, *P* = 0.705). The amounts of dengue virus in the mosquito midguts, heads, and salivary glands were determined by RT-qPCR. The average number of dengue RNA copies (log_10_) in these tissues for each population at 14 dpi are shown in **Figure 1** and **Table 3**. The numbers of dengue RNA copies in the midguts, heads and salivary glands are no apparent differences among the populations, except for a certain tissue in individual populations. The infection rate for each tissue is not correlated with viral load for the eight *Ae. albopictus* populations (midguts: *r* = 0.130, *P* = 0.759; heads: *r* = 0.209, *P* = 0.620; salivary glands: *r* = 0.520, *P* = 0.186). The numbers of dengue RNA copies in tissues of the mosquitoes from southern-western China (MS, GZ, ZJ, LS) are lower than those from eastern-central-northern China (BJ, SJZ, HZ, WH) (midguts: t = 2.503, *P* = 0.013; heads: t = 2.579, *P* = 0.011; salivary glands: t = 3.800, *P* < 0.001).

**Table 2:** Rates of dengue virus infection, dissemination, potential transmission, and population potential transmission by *Ae. albopictus* females from eight different populations.

Populations	IR	MIR	DR	TR	PTR
BJ	80.00% (32/40)	80.00% (24/30)	91.67% (22/24)	70.83% (17/24)	56.67% (17/30)
SJZ	82.50% (33/40)	83.33% (25/30)	84.00% (21/25)	68.00% (17/25)	56.67% (17/30)
HZ	87.50% (35/40)	96.67% (29/30)	75.86% (22/29)	62.07% (18/29)	60.00% (18/30)
WH	82.50% (33/40)	86.67% (26/30)	76.92% (20/26)	73.08% (19/26)	63.33% (19/30)
MS	85.00% (34/40)	93.33% (28/30)	71.43% (20/28)	60.71% (17/28)	56.67% (17/30)
GZ	75.00% (30/40)	80.00% (24/30)	83.33% (20/24)	70.83% (17/24)	56.67% (17/30)
ZJ	77.50% (31/40)	76.67% (23/30)	69.57% (16/23)	52.17% (12/23)	40.00% (12/30)
LS	90.00% (36/40)	93.33% (28/30)	82.14% (23/28)	50.00% (14/28)	46.67% (14/30)

IR, infection rate = no. infected mosquitoes/no. tested mosquitoes (%); MIR, midgut infection rate = no. infected midguts/no. tested midguts (%); DR, dissemination rate = no. infected heads/no. infected midguts (%); TR, potential transmission rate = no. infected salivary glands/no. infected midguts (%); PTR, potential population transmission rate = no. infected salivary glands/no. tested midguts (%).

**Table 3:** Correlation of *w*AlbA and *w*AlbB with DENV-2 loads in the tissues and whole mosquitoes for *Ae. albopictus* populations in China.

Populations	Midguts^m^	Heads^m^	Salivary glands^m^	Mosquitoes^n^	*w*AlbA^n^	*w*AlbB^n^
BJ	4.704	4.114	3.717	1.011	0.036	0.629
SJZ	5.314	4.303	3.747	0.878	0.093	0.760
HZ	5.109	4.006	3.640	0.943	0.091	0.702
WH	5.077	4.683	3.925	1.065	0.044	0.382
MS	4.694	3.855	3.208	0.827	0.064	0.810
GZ	4.888	4.170	3.426	0.748	0.105	0.920
ZJ	4.537	3.947	3.327	0.710	0.055	0.837
LS	4.556	3.832	3.590	0.791	0.058	0.798
r^a^	0.520	-0.067	-0.192	-0.393		
*P*_a_ *-* value	0.187	0.875	0.650	0.336		
r^b^	-0.354	-0.729^*^	-0.785^*^	-0.909^*^		
*P*_b_ *-* value	0.390	0.040	0.021	0.002		

^m^ Mean DENV-2 copies (log_10_) in infected midguts, heads, and salivary glands of Ae. albopictus from the eight populations.

^n^ Mean loads of DENV-2 and Wolbachia in whole mosquitoes relative to the Rps6 gene.

r^a^: Pearson’s correlation coefficient between wAlbA and DENV-2 in the tissues and whole mosquitoes. P_a_ value: P value corresponding to r^a^. r^b^: Pearson’s correlation coefficient between wAlbB and DENV-2 in tissues and whole mosquitoes. P_b_ value: P value corresponding to r^b^.

*P < 0.05.

### Effects of Immune-Associated Genes on Vector Competence of *Ae. albopictus*

**Figure 2:**
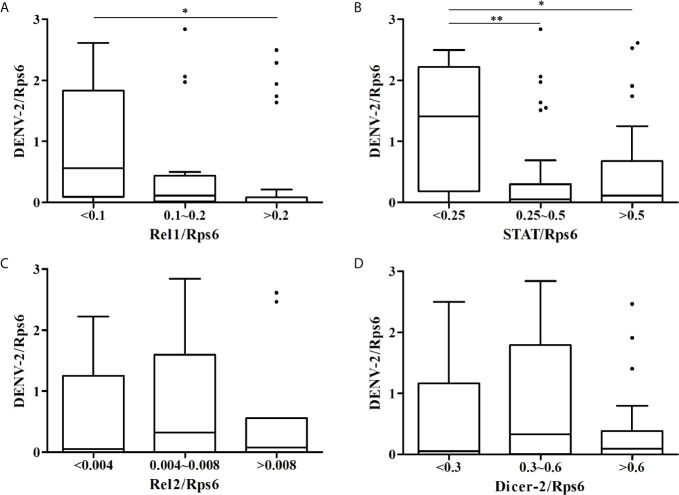
Boxplots showing DENV-2 loads among the different expression levels of immune-associated genes [Rel1 **(A)**, STAT **(B)**, Rel2 **(C)**, Dicer-2 **(D)**] in whole mosquitoes from the eight *Ae. albopictus* populations. *P < 0.05, **P < 0.01.

**Figure 3:**
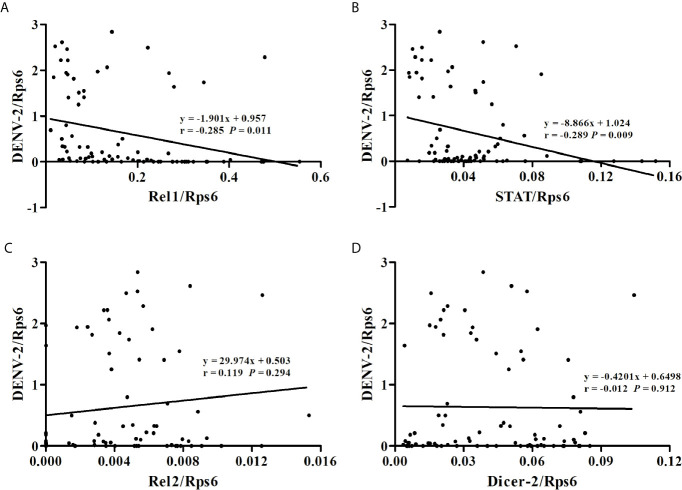
Correlations between DENV-2 loads and the expression levels of immune-associated genes [Rel1 **(A)**, STAT **(B)**, Rel2 **(C)**, Dicer-2 **(D)**] in whole mosquitoes from the eight *Ae. albopictus* populations.

At 14 dpi, the DENV-2 loads and expression levels of immune-associated genes in whole mosquitoes from the eight *Ae. albopictus* populations were determined by RT-qPCR as normalized using the host Rps6 gene (**Table S2**). As shown in **Figure 2**, the mosquitoes with high expression level of Rel1 or STAT gene have relatively lower DENV-2 loads. The DENV-2 loads in whole mosquitoes have a negative correlation with the expression levels of the Rel1 gene (*r* = -0.285, *P* = 0.011) and STAT gene (*r* = -0.289, *P* = 0.009) (**Figure 3**). The DENV-2 loads are not correlated with the expression levels of the Rel2 gene (*r* = 0.119, *P* = 0.294) or Dicer2 gene (*r* = -0.012, *P* = 0.912). At the same time, the DENV-2 loads in whole mosquitoes from the populations (MS, GZ, ZJ, LS) in southern-western China have a negative correlation with the expression levels of the Rel1 gene (*r* = -0.361, *P* = 0.022) and STAT gene (*r* = -0.335, *P* = 0.034), while the DENV-2 loads in whole mosquitoes from the populations (BJ, SJZ, HZ, WH) in eastern-central-northern China are not correlated with the expression levels of the Rel1 gene (*r* = -0.185, *P* = 0.254) and STAT gene (*r* = -0.278, *P* = 0.083).

### Effects of *Wolbachia* Strain on Vector Competence of *Ae. albopictus*

The relative densities of the *w*AlbA and *w*AlbB strains were measured for individual females sampled from the eight *Ae. albopictus* populations, and the results are shown in **Table 3**. The data were normalized using the host Rps6 gene, which also allows the densities of the two *Wolbachia* strains to be compared between different adult sizes.

**Figure 4:**
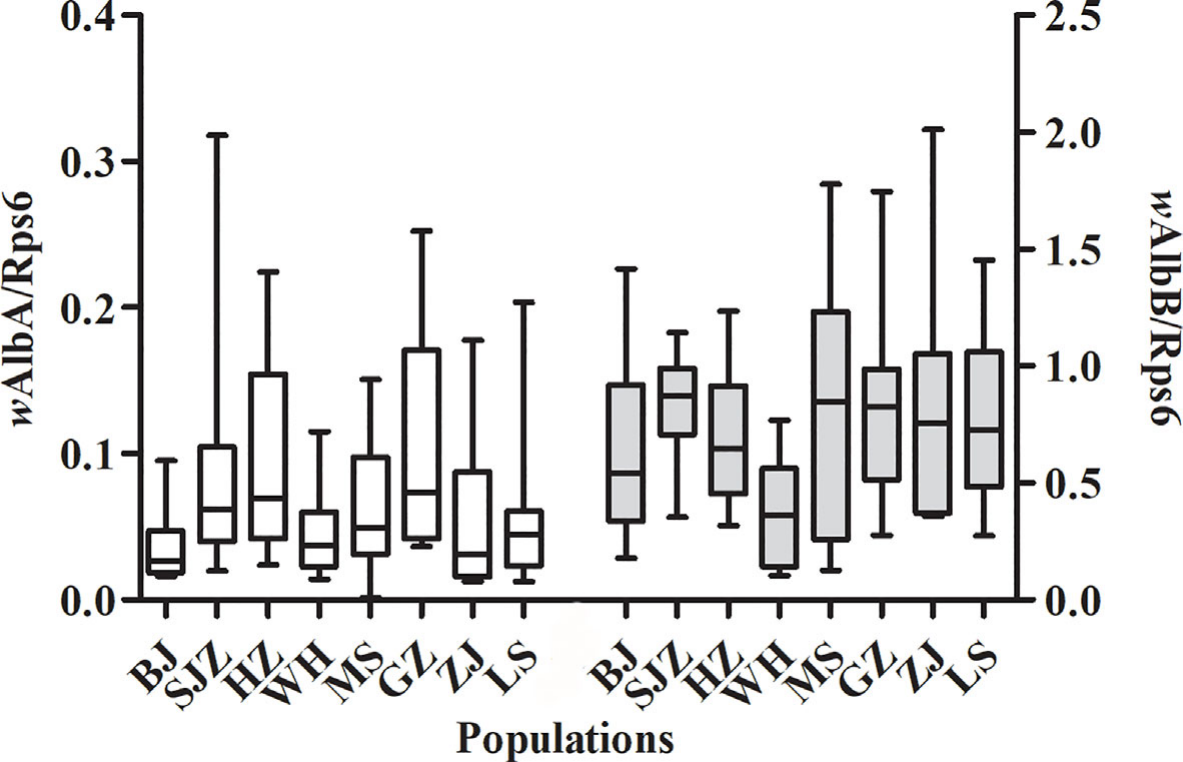
Relative densities of the *w*AlbA and *w*AlbB strains in *Ae. albopictus* from the eight populations in China.

**Figure 4** shows a higher density of *w*AlbB than that of *w*AlbA, and this difference is significant in the eight *Ae. albopictus* populations (t = -12.731, *P* < 0.001). There is no correlation between the densities of the *w*AlbA and *w*AlbB strains (*r* = 0.532, *P* = 0.174). There was not significant correlation between the relative density of the *w*AlbA/B strain and the expression levels of immune-associated genes in mosquitoes (**Table S3**). The relative density of the *w*AlbB strain in mosquitoes from southern-western China (MS, GZ, ZJ, LS) are higher than those from eastern-central-northern China (BJ, SJZ, HZ, WH) (t = 2.380, *P* = 0.020). The relative density of the *w*AlbB strain shows a negative correlation with the mean loads of DENV-2 in the heads (*r* = -0.729, *P* = 0.040), salivary glands (*r* = -0.785, *P* = 0.021), and whole mosquitoes (*r* = -0.909, *P* = 0.002).

## Discussion

The vector competence for DENV-2 among different geographic strains of *Ae. albopictus* populations is influenced by ecological structure, climate, virus titer, endosymbiont bacteria, and genetic factors. Accordingly, in the present study, different geographic strains of *Ae. albopictus* were collected and infected with the same titer of DENV-2 in the same environment to assess their vector competence, eliminating the effects of environment, climate, and virus titer. We further explored the effects of genetic factors and endosymbiont bacteria among different geographic strains of *Ae. albopictus* populations.

The infection rates for each tissue (IR, MIR, DR, TR and PTR) are not significantly different and not correlated with DENV-2 loads in the eight *Ae. albopictus* populations. This non-correlation has been reported in other studies (Bosio et al., 1998; Lambrechts et al., 2009). There are no apparent differences in the numbers of dengue RNA copies in the tissues among different geographic strains of *Ae. albopictus* populations in China. A possible explanation for this result is that they share genetic background. Gao et al. (2021) reported that four major migration trends which were observed among the different climatic regions with high gene flow contributed to the similarity of *Ae. albopictus* populations in China. Our previous study (Wei et al., 2019) also reported that the strong gene flow assisted by human activities inhibited population differentiation of *Ae. albopictus* populations in China, and the 34 populations across China were assigned to two genetic clusters with very low genetic differentiation (southern-western cluster and eastern-central-northern cluster). The low differentiation may be attributed to the current adaptation of *Ae. albopictus* population with shared genetic background to the ecological environment of the new migration places before mosquito collection, including local climate and microbial infection.

Although the difference in DENV-2 loads between *Ae. albopictus* populations is not obvious, the midguts, heads, and salivary glands in mosquitoes from dengue fever endemic areas in southern-western China infected with DENV-2 have lower loads relative to the populations from eastern-central-northern China, which may be related to the evolution of mosquitoes in these areas upon frequent virus infection. Our previous study (Wei et al., 2019) also indicated that the genetic diversity of *Ae. albopictus* in dengue fever endemic areas is higher than that in non-endemic areas in northern China. Local adaptation is predicted when the pathogen has an evolutionary advantage over the host, such as higher mutation rate, shorter generation time, higher migration rate, and larger population size (Gandon and Van Zandt, 1998; Kaltz and Shykoff, 1998; Kawecki and Ebert, 2004). So the *Ae. albopictus* populations from southern-western China could have slightly stronger immune defenses against dengue virus, which may be associated with that the host from southern-western China might have an evolutionary advantage over that from eastern-central-northern China.

We also found that the DENV-2 loads in whole mosquitoes show a negative correlation with Rel1 gene and STAT gene expression, which is related to immune regulation of mosquitoes to DENV-2. The expression of Rel1 gene and STAT gene show a negative correlation with the DENV-2 loads in whole mosquitoes from southern-western China but not those from eastern-central-northern China, which may support the claim of stronger immune defenses in the mosquitoes from southern-western China. The transcription factor Rel1 translocates from the cytoplasm to the nucleus and binds to κB motifs on the promoters of many antimicrobial peptides, such as diptericin and cecropins, that are active against pathogenic microorganisms (Shin et al., 2006; Luplertlop et al., 2011). Some studies have reported that Rel1 and its downstream antimicrobial peptides are upregulated to control infection against DENV upon infection (Xi et al., 2008; Sim and Dimopoulos, 2010). Rel1 is also regulated by many factors in mosquitoes to antagonize the replication and dissemination of dengue virus (Xi et al., 2008; Hussain et al., 2013).

The signal transducers and activators of transcription (STAT) are recruited by the Dome/Hop complex, phosphorylated, and dimerized, then translocate to the nucleus where they activate the transcription of specific effector genes, such as the virus-induced RNA 1 (*vir*-1) gene, that play roles in antiviral immunity (Dostert et al., 2005; Souza-Neto et al., 2009). Arboviruses, including Japanese encephalitis virus (JEV), West Nile virus (WNV), and DENV, have been shown to interfere with the JAK-STAT pathway by inhibiting JAK phosphorylation, thereby preventing translocation of STAT to the nucleus and/or altering the expression of IFN-stimulated genes (Lin R. J. et al., 2004; Guo et al., 2005; Ho et al., 2005). It has been reported that STAT phosphorylation and DNA binding activity are inhibited in C6/36 mosquito cells infected with JEV (Lin C. C. et al., 2004). The JAK-STAT pathway has been shown to mediate increased resistance to DENV in infected *Ae. aegypti* (Souza-Neto et al., 2009).

*Wolbachia pipientis* are symbiotic bacteria, vertically transmitted from mother to offspring, of which *w*AlbA and *w*AlbB exist naturally in *Ae. albopictus* populations (Tsai et al., 2017; Mohanty et al., 2019; Wei et al., 2019). Some types of *Wolbachia* have been introduced into mosquitoes that are not natural host of *Wolbachia* as a means to limit their ability to transmit important arboviruses including DENV, CHIKV, and ZIKV (Xi et al., 2005; Moreira et al., 2009; Bian et al., 2013). Several countries, including Brazil, Australia, and Vietnam, have participated in field trials of DENV control based on the release of *Wolbachia*-infected mosquitoes into the wild (World Mosquito Program, 2017). Furthermore, *Wolbachia*-infected mosquitoes from the same field populations maintain reduced susceptibility to DENV under laboratory conditions (Frentiu et al., 2014). A very low *Wolbachia* density could contribute to absence of *Wolbachia*-mediated resistance to dengue virus in *Ae. albopictus* (Lu et al., 2012). The density-dependent inhibition to DENV of *Wolbachia* in mosquito vectors may directly influence the disease transmission in the field. Therefore, it is very important to detect *Wolbachia* density of field populations to evaluate the vector competence of *Ae. albopictus.*

In the present study, we found that the *w*AlbB strain is present at a higher density than that of the *w*AlbA strain in *Ae. albopictus*, which is consistent with the results of previous studies (Dutton and Sinkins, 2004; Hu et al., 2020). The relative density of the *w*AlbB strain in mosquitoes from southern-western China (MS, GZ, ZJ, LS) are higher than those from eastern-central-northern China (BJ, SJZ, HZ, WH). Furthermore, the DENV-2 loads in whole mosquitoes, heads, and salivary glands show a negative correlation with the relative density of *w*AlbB *Wolbachia*. These results indicate that the density of *w*AlbB *Wolbachia* may have changed due to the influence of the habitat environment before mosquito collection (Wiwatanaratanabutr and Kittayapong, 2009; Rosso et al., 2018; Thongsripong et al., 2018; Hu et al., 2020), and then affect the DENV-2 loads of local mosquito population. Xue et al. (2018) quantified the impact of *w*AlbB *Wolbachia* in reducing the transmission of CHIKV, DENV, and ZIKV in *Ae. albopictus* and *Ae. aegypti*. Stable infections of *w*AlbB *Wolbachia* were established and found to inhibit the replication and dissemination of DENV in *Ae. aegypti* (Xi et al., 2005; Bian et al., 2010). *w*AlbB *Wolbachia* downregulate the expression of two putative mosquito DENV receptors, knockdown of which results in the inhibition of DENV-2 binding to mosquito cells (Lu et al., 2020). Furthermore, in Malaysia, the incidence of dengue was reduced at release sites with high endemic dengue transmission after *Ae. aegypti* mosquitoes carrying *w*AlbB were released and maintained at high population frequency (Nazni et al., 2019).

In this study, the loads of DENV-2 in *Ae. albopictus* showed negative correlation with Rel1 gene expression, STAT gene expression, and *w*AlbB density, but not with Rel2 gene expression, Dicer2 gene expression, and *w*AlbA density. However, this does not mean that Rel2, Dicer2, and *w*AlbA do not play antiviral roles in *Ae. albopictus* infected with DENV-2. They may have large, small, or similar degrees of antiviral effect in *Ae. albopictus* (Antonova et al., 2009; Lambrechts et al., 2013; Kumar et al., 2018; Chouin-Carneiro et al., 2020), but there is no difference in these factors among different mosquito individuals or populations. No significant correlation was observed between the density of *Wolbachia* strains and the expression of immune-associated genes. One possible reason might be that the naturally stable infection of *Wolbachia* in mosquitoes may have been in a state of balance, affecting the comprehensive and complex immune chain reaction rather than a specific pathway. The other one might be that the density of natural *Wolbachia* strains and expression of immune-associated genes in mosquitoes with different immune defense capabilities may be affected by individual and population differences. When introduced to nonnative mosquito hosts, *Wolbachia* infection can enhance the immune response and increase the expression of reactive oxygen species (ROS) and Rel1 (Pan et al., 2012). The effect of other immune markers on the immune response of mosquitoes to dengue virus will be worthy of our next study besides Rel1 and STAT, such as ROS (Wang et al., 2019) and nitric oxide (NO) (Ramos-Castañeda et al., 2008).

To verify the specific roles and differences of these immune genes and pathways in different mosquito populations, different experimental approaches are required, such as setting more infection time points, interfering with immune genes, and improving immune response. Such are the limitations of this study. Another big limitation is that the change in environment and climate from fields to laboratory may affect the immune respond and viral loads of *Ae. albopictus.* In this regard, in the next study we will simulate the field environment to conduct the experiments and comprehensively discuss the influence of the field environment and immune system on the vector competences of local mosquitoes. Nevertheless, this study provides a preliminary understanding of vector competence and immune levels of *Ae. albopictus* populations in different regions of China.

## Conclusion

This study investigated not only the vector competences for DENV-2 of different *Ae. albopictus* populations in China, but also the factors relating to innate immunity that influence mosquito susceptibility to DENV-2. The amounts of DENV-2 in the mosquito midguts, heads, and salivary glands from southern-western China are lower than those from eastern-central-northern China. Furthermore, the differences in vector competence may be related to the innate immunity of mosquitoes involving Rel1 in the Toll pathway, STAT in the JAK-STAT pathway, and the relative density of the *w*AlbB strain. The data gained from this study provide useful information for the epidemiology, prevention, and control of vector-borne diseases.

## Data Availability Statement

The original contributions presented in the study are included in the article/**Supplementary Material**. Further inquiries can be directed to the corresponding author.

## Author Contributions

YW and XZ conceived and designed the experiments. YW, JW, Y-HW, ZS, KH, and YC performed the experiments. YW and Y-HW analyzed the data. YW, XZ, DZ, and GZ wrote and revised the manuscript. All authors contributed to the article and approved the submitted version.

## Funding

This work was supported by the National Natural Science Foundation of China (31630011), Natural Science Foundation of Guangdong Province (2017A030313625), and the Science and Technology Planning Project of Guangzhou (201804020084).

## Conflict of Interest

The authors declare that the research was conducted in the absence of any commercial or financial relationships that could be construed as a potential conflict of interest.
